# Closing the gaps for animal seed dispersal: Separating the effects of habitat loss on dispersal distances and seed aggregation

**DOI:** 10.1002/ece3.3113

**Published:** 2017-06-12

**Authors:** Landon R. Jones, Scott M. Duke‐Sylvester, Paul L. Leberg, Derek M. Johnson

**Affiliations:** ^1^ Department of Biology University of Louisiana at Lafayette Lafayette LA USA; ^2^ Department of Biology Virginia Commonwealth University Richmond VA USA

**Keywords:** fragment entrapment, individual‐based model, long‐distance dispersal, mechanistic model, spatial pattern, spatially contagious dispersal

## Abstract

Habitat loss can alter animal movements and disrupt animal seed dispersal mutualisms; however, its effects on spatial patterns of seed dispersal are not well understood. To explore the effects of habitat loss on seed dispersal distances and seed dispersion (aggregation), we created a spatially explicit, individual‐based model of an animal dispersing seeds (SEADS—Spatially Explicit Animal Dispersal of Seeds) in a theoretical landscape of 0%–90% habitat loss based on three animal traits: movement distance, gut retention time, and time between movements. Our model design had three objectives: to determine the effects of (1) animal traits and (2) habitat loss on seed dispersal distances and dispersion and (3) determine how animal traits could mitigate the negative effects of habitat loss on these variables. SEADS results revealed a complex interaction involving all animal traits and habitat loss on dispersal distances and dispersion, driven by a novel underlying mechanism of fragment entrapment. Unexpectedly, intermediate habitat loss could increase dispersal distances and dispersion relative to low and high habitat loss for some combinations of animal traits. At intermediate habitat loss, movement between patches was common, and increased dispersal distances and dispersion compared to continuous habitats because animals did not stop in spaces between fragments. However, movement between patches was reduced at higher habitat loss as animals became trapped in fragments, often near the parent plant, and dispersed seeds in aggregated patterns. As movement distance increased, low time between movements and high gut retention time combinations permitted more movement to adjacent patches than other combinations of animal traits. Because habitat loss affects movement in a nonlinear fashion under some conditions, future empirical tests would benefit from comparisons across landscapes with more than two levels of fragmentation.

## 1 INTRODUCTION

Over 60% of temperate and over 80% of tropical tree species exhibit adaptations for animal seed dispersal (Howe & Smallwood, 1982). The spatial patterns of seed deposition in this mutualism are shaped by the movements, behaviors, and physiology of animal dispersers (Karubian & Durães, 2009; Nathan & Muller‐Landau, 2000; Schupp, Milleron, & Russo, 2002). Two main measures of these spatial patterns that have important consequences for plant recruitment are long‐distance and aggregated seed dispersal (Nathan et al., 2008; Schupp et al., 2002). Dispersal distances, or the distance seeds are moved from parent plants, can include rare long‐distance dispersal events that often determine range expansion, gene flow among populations, and colonization of new habitats for plant species (reviewed in Nathan et al., 2008). Restriction of dispersal distances and long‐distance events in particular can have important and often cryptic consequences for plant demography, such as low gene flow across a landscape (e.g., Hamrick, Murawski, & Nason, 1993) and eventual extirpations of plant species from isolated forest fragments (e.g., Guimarães, Galetti, & Jordano, 2008). Aggregated seed dispersal across a landscape can increase small‐scale competition and attract seed predators, often increasing seed mortality (Garzón‐López et al., 2015; Kwit, Levey, & Greenberg, 2004; Russo & Augspurger, 2004; Schupp et al., 2002). Highly aggregated dispersal can have negative consequences for tree populations, such as reduced sapling recruitment (e.g., Harrison et al., 2013), and can cascade through all life stages, decreasing population sizes by as much as 10‐fold (Caughlin et al., 2015).

Habitat loss and the resulting fragmentation is a major anthropogenic factor that can alter and disrupt seed dispersal mutualisms (Cordeiro & Howe, 2003; Rodríguez‐Cabal, Aizen, & Novaro, 2007; reviewed in McConkey et al., 2012). Habitat loss has been shown to alter the geometry of disperser movements, which may, in turn, affect spatial patterns of seed dispersal (e.g., Levey, Bolker, Tewksbury, Sargent, & Haddad, 2005; Uriarte et al., 2011). However, the effects of habitat loss on spatial patterns of animal‐mediated seed dispersal and its driving mechanisms are not well understood (Markl et al., 2012; McConkey et al., 2012). A few studies have found evidence for reduced dispersal distances in fragmented habitats (reviewed in McConkey et al., 2012); however, the effects of habitat loss on aggregated seed dispersal have been virtually unexplored. In contrast, studies of animal‐mediated pollen dispersal suggest that habitat loss typically does not limit pollen movement and often increases long‐distance dispersal (reviewed in Hamrick, 2010), even if disperser movements are restricted (i.e., Volpe, Robinson, Frey, Hadley, & Betts, 2016). Thus, it is not well understood how the effects of habitat loss on the movements of seed dispersers translate to changes in seed dispersal or whether they depend on interactions with the landscape and other variables.

In this paper, we focus on the mechanism of how habitat loss alters animal movements and its subsequent consequences for seed dispersal distances and aggregation across the landscape. To close knowledge gaps and systematically explore these effects, we created a spatially explicit, mechanistic model of an animal dispersing seeds in a theoretical landscape (SEADS—Spatially Explicit Animal Dispersal of Seeds). SEADS allows us to simultaneously assess how the values of three important disperser traits, movement distance, gut retention time, and time between movements (e.g., Levey et al., 2005; Murray, 1988; Spiegel & Nathan, 2007; Uriarte et al., 2011), interact with habitat loss and each other within the same framework, according to three objectives. Our first objective was to explore how animal traits affect seed dispersal and aggregation. Our second objective was to explore the effects of habitat loss on seed dispersal and aggregation. Our third objective was to determine how animal traits could mitigate the potential negative effects of habitat loss on seed dispersal and aggregation. Identifying and quantifying the interactions of habitat loss and animal traits that facilitate high seed dispersal distances and low aggregation can help conservationists predict which animal species or guilds would be effective seed dispersers in landscapes affected by habitat loss.

## 2 MATERIALS AND METHODS

### 2.1 Study design

**Figure 1:**
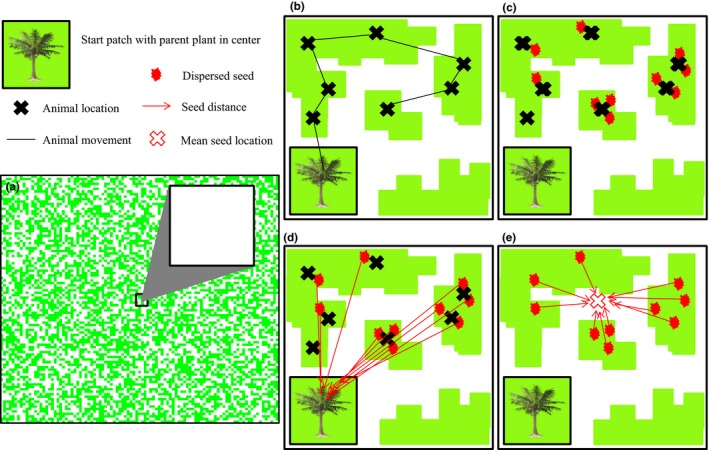
Illustration of model processes for SEADS, an individual‐based model of an animal dispersing seeds in a landscape of increasing habitat loss. (a) Example of one of 10 landscapes experiencing 0%–90% habitat loss (50% shown). Green represents suitable habitat, and white represents matrix (unsuitable habitat). The animal begins at the parent plant in a start patch 100 m² in the middle of the landscape after consuming 100 seeds. (b) The animal moves within and among suitable habitat according to movement distance and time between movements. (c) One or multiple seeds are dispersed according to gut retention times at or near animal locations. (d) Dispersal distances are calculated from each seed to the parent plant in the middle of the start patch. (e) Seed dispersion (aggregation) is calculated as the mean distance from each seed to the mean location of all seeds

To simulate an animal dispersing seeds in a theoretical landscape experiencing various levels of habitat loss (Figure 1a), we created SEADS (Spatially Explicit Animal Dispersal of Seeds) in program R (R Development Core Team 2015). In SEADS, the movement of an animal disperser is simulated within and among suitable habitat fragments according to two animal traits, a movement distance probability distribution and time between movements (Figure 1b). As the animal moved within the landscape, gut retention times determined when seeds were dispersed and seed locations were recorded at or near animal locations (Figure 1c). Note the distinction between mean movement distances, which is a trait intrinsic to the animal independent of landscape effects, while seed dispersal distance (hereafter dispersal distance) is the resulting distance of an individual seed from the parent plant when animal movement is simulated on theoretical landscapes (Figure 1d). The metric for seed dispersion, our metric of seed aggregation, was calculated by measuring the distance of individual seeds to the mean of all seed locations (dispersion, Figure 1e). Additionally, the data generated from model simulations were analyzed to assess the relative importance of the parameters (habitat loss and animal traits) and their interactions in shaping metrics of dispersal distance and seed dispersion.

### 2.2 Model landscape

**Figure 2:**
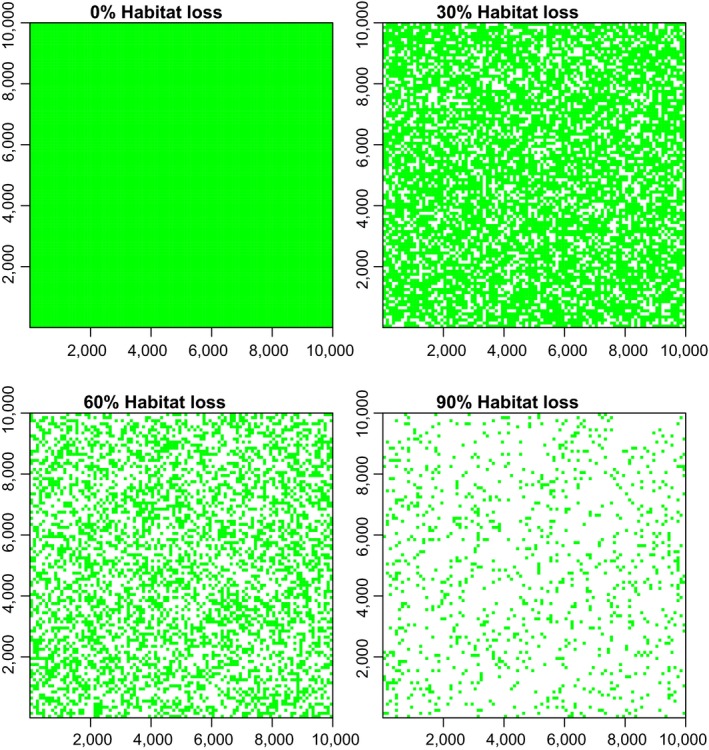
Configurations for 4 of 10 theoretical landscapes representing levels of increasing habitat loss from 0%–90% in 10% increments. Green cells represent suitable habitat, white cells represent matrix (unsuitable habitat) for a simulated animal disperser. Each landscape represents a square of 10,000 m² or 100 ha

The landscape of SEADS represented a continuous block of suitable habitat consisting of 10,000 × 10,000 cells, with each cell representing 1 × 1 m, for a total of 10,000 ha or 100 km². Habitat loss was modeled in 10 configurations as a percentage of the total landscape area converted to unsuitable habitat (matrix) with the following treatment levels: 0%, 10%, 20%, 30%, 40%, 50%, 60%, 70%, 80%, 90% (Figure 2). In each configuration, the landscape began as one continuous block of suitable habitat. For each configuration, we then increased habitat loss by randomly selecting 1 ha squares (100 m²) within the landscape and converting them to matrix (unsuitable habitat) until reaching the desired treatment levels (0%–90%) for respective configurations (Figure 2). Suitable habitat cells were assigned a value of 1, and cells in unsuitable habitat were assigned a value of 0. In each scenario, the animal began in the middle of a start patch consisting of 100 m² suitable habitat in the middle of the landscape. This start point represented a fruiting tree from which the animal obtained 100 seeds.

### 2.3 Parameter estimation

Some traits of animal vectors may mitigate the potentially negative consequences of habitat loss on seed dispersal distance and dispersion. To model the effects of habitat loss on our seed dispersal metrics, we parameterized SEADS simulations with three animal traits that have been shown as influential drivers of dispersal distances and could presumably affect seed dispersion. The distance a disperser moves within a given time period (movement distance), and the time seeds are retained in the gut before regurgitation or defecation (gut retention time) has been quantified to characterize and compare different animal species for dispersal distance (e.g., Holbrook & Loiselle, 2007; Murray, 1988; Spiegel & Nathan, 2007; Uriarte et al., 2011). The time interval between movements (Murray, 1988), residence time (Sun, Ives, Kraeuter, & Moermond, 1997; Wotton & Kelly, 2012), and perching times (Levey et al., 2005; Morales & Carlo, 2006; Uriarte et al., 2011), or how often an animal moves within a fixed time period, hereafter time between movements, also affects dispersal distance (e.g., Kays, Jansen, Knecht, Vohwinkel, & Wikelski, 2011; Westcott, Bentrupperbäumer, Bradford, & McKeown, 2005).

### 2.4 Review of the animal seed dispersal literature

We reviewed studies from the animal seed dispersal literature through 2016 to construct ranges of informative and plausible values for the three animal traits: movement distance, gut retention time, and time between movements. Our goal was to find data that spanned the range of values for vertebrate seed dispersal for which dispersal distances were available and not to complete an exhaustive review of all values reported for our traits of interest (e.g., Côrtes & Uriarte, 2013; Wotton & Kelly, 2012). Thus, among the studies we reviewed, we selected 19 species from 15 studies representing a wide range of body sizes and taxa, to capture the range of plausible values from available data for each animal trait that would also be informative for SEADS (Table 1). When multiple values were provided for parameters, such as dispersal distances for different plant species for a given animal disperser (e.g., Murray, 1988) or time between movements for the same disperser during foraging or nonforaging periods (Sun et al., 1997), a mean value was determined across appropriate values. For each animal trait, a scale of doubling parameter values from low to high was created based on the range of empirical values obtained from this literature review (Table 1).

**Table 1:** Average values for three animal traits, movement distance (MD), gut retention time (GRT), and time between movements (TBM), and corresponding seed dispersal distances (SDD) for 19 animal species across taxa and body sizes, taken from literature studies. MD and GRT values were either reported separately, or together as the cumulative distance animals moved (CD) over a fixed time interval (TI)

Mass (kg)	Animal	MD (m)	CD (m) in	TI (min)	GRT (min)	TBM (min)	SDD (m)	References
0.008	Lizard	–	72	3,600	2,640	–	72	Rodríguez‐Pérez et al. (2012)
0.030^a^	Songbird	17.0	–	–	45	2.55^b^	200	Levey et al. (2005, 2008)
0.033^a^	Songbird	58.7	–	–	19	8.58	50–60	Murray, 1988;
0.041	Songbird	20.2	75	43	35	3.23^c^	302	Spiegel and Nathan (2007)
0.056^a^	Songbird	61.3	–	–	17	10.73	50–60	Murray (1988)
0.062^a^	Songbird	38.8	–	–	22	9.80	50–60	Murray (1988)
0.119	Songbird	48.5	154	36	135	3.65^c^	1,168	Spiegel and Nathan (2007)
0.250	Turaco	44.1	–	–	70	22	138	Sun et al. (1997)
0.288	Toucan	–	200	15	28	–	100–200	Holbrook and Loiselle (2007)
0.566	Toucan spp	–	100	15	34	–	>100	Holbrook and Loiselle (2007)
0.635	Hornbill	–	630	15–150	57	–	512	Lenz et al. (2011)
0.650	Pigeon	77	–	–	120	27.02	85	Wotton et al. (2012)
0.884^a^	Marten	–	133	0–60	261	–	507	Hickey, Flynn, Buskirk, Gerow, and Willson (1999)
5.4^a^	Monkey	–	845	1,180	1,180	–	317	Yumoto et al. (1999)
7.4^a^	Monkey	–	486	376	376	–	327	Yumoto et al. (1999)
8.2^a^	Tortoise	–	229	11,952	11,952	–	224	Jerozolimski et al. (2009)
63	Cassowary	–	3,946	32,832	433	–	336	Westcott et al. (2005)
250	Tortoise	–	394	17,280	17,280	–	394	Blake et al. (2012)
2,840	Elephant	–	1,988	6,960	2,370	–	1,522	Campos‐Arceiz et al. (2008)

a Not given in reference, see additional reference in Appendix S2.

b Base perching time, modified further by distance from edge.

c Calculated from data.

### 2.5 Movement distance

**Figure 3:**
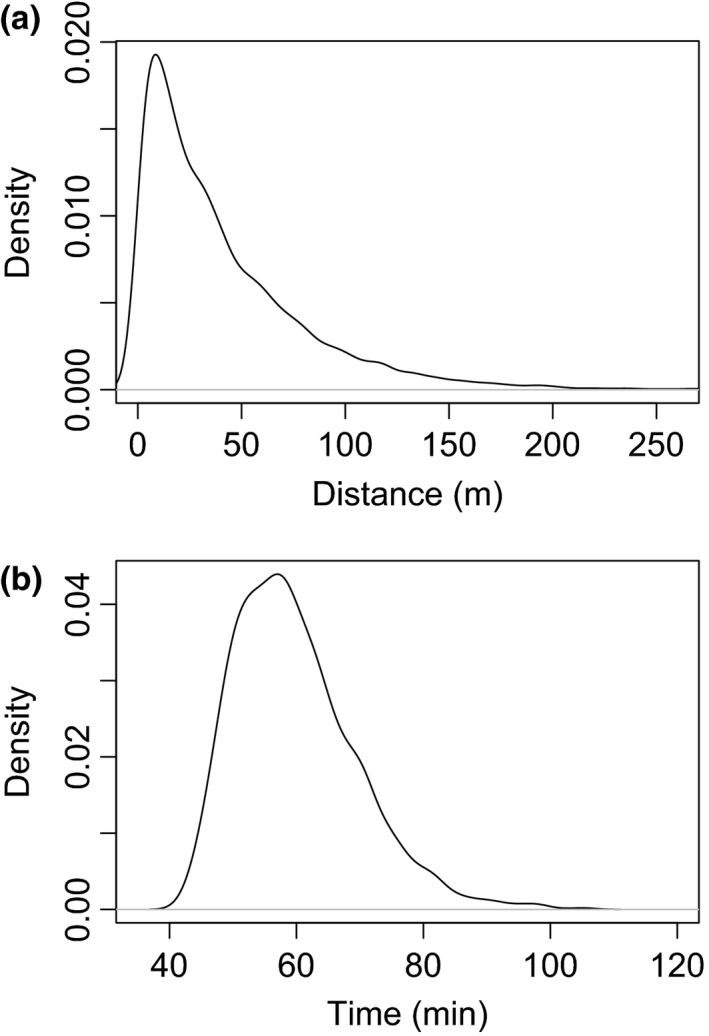
Exponential distribution from which values for animal movement distances were drawn to simulate the movement of an animal seed disperser (a). Mean movement distances ranged from 5–160 m in six treatment levels (40 m shown, drawn from 10,000 samples). Discretized gamma distribution (shape = 4, scale = 5) of whole minutes from which 100 gut retention times were drawn (b). Mean gut retention times ranged from 15–480 min in six treatment levels (60 min shown, drawn from 10,000 samples)

Mean distance the animal moved (MD) was sampled from an exponential distribution (Figure 3a) with its mean set to one of six treatment levels: 5, 10, 20, 40, 80, and 160 m. Each time the animal moved, a new random distance was selected. MD was defined as the distance the animal moved from consecutive locations and as one movement length (e.g., Levey et al., 2005) or step length (Cousens, Hill, French, & Bishop, 2010), as reported in four main studies (Murray, 1988; Levey et al., 2005; Spiegel & Nathan, 2007; Wotton & Kelly, 2012; Table 1). These disperser studies separated MD from gut retention time (GRT) and reported values of MD for six small to medium passerines and one pigeon species, with distances ranging from 17.0–77 m (Table 1). In the absence of direct information, other values on our MD scale were established to cover the range of disperser sizes and movements from low (8 g lizards moving 72 m within a GRT of 2–3 days, Rodríguez‐Pérez, Larrinaga, & Santamaría, 2012) to high (2,000+ kg elephants moving 1,988 m within a GRT of 5 days, Campos‐Arceiz et al., 2008; Table 1).

### 2.6 Gut retention time

Parameter values for gut retention time were sampled from a gamma distribution (Figure 3b) with means of 15, 30, 60, 120, 240, and 480 min. The scale and shape of our gamma distribution (shape = 4, scale = 5) was set to produce a fat‐tailed distribution, characteristic of observed distributions of gut retention times (e.g., Levey et al., 2005; Morales & Carlo, 2006). Although some animals can retain seeds as long as 1–12 days (e.g., Campos‐Arceiz et al., 2008; Jerozolimski, Ribeiro, & Martins, 2009; Blake et al., 2012; Rodríguez‐Pérez et al., 2012; Table 1), our scale was limited to 8 hr or less to represent the peak range of potential daily hours when animals are typically awake and can disperse seeds. Seeds from fruits consumed later in the afternoon, for example, are most likely deposited at sleeping sites in the morning after waking if the retention time falls during sleeping periods (e.g., Russo, Portnoy, & Augspurger, 2006; Yumoto, Kimura, & Nishimura, 1999).

### 2.7 Time between movements

Time between movements was deterministic and evenly spaced at one of six durations: 4, 8, 16, 32, 64, and 128 min. To create this scale, the examples found for this parameter were all avian, from four studies of small and medium passerine ranging from 2.6 to 10.7 min between movements (Murray, 1988; Levey et al., 2005; Spiegel & Nathan, 2007; pers comm, Orr Spiegel, Table 1) and a large pigeon species with a mean of 27.02 min between movements (Wotton & Kelly, 2012). However, many animal dispersers are less active during some portion of the day (e.g., Kays et al., 2011; Russo et al., 2006; Westcott et al., 2005) and may spend up to two (e.g., Rodríguez‐Pérez et al., 2012) or rarely up to 5 hr or more (Wotton & Kelly, 2012) at rest between movements.

### 2.8 Model processes

#### 2.8.1 Animal movements

Each simulation in SEADS began with an animal that had consumed 100 seeds at time 0 in the center of the start patch (Figure 1a). The temporal component of the model was divided into discrete 1 minute time steps and each run of the model ended when the animal dispersed 100 seeds. Time between movements was set to a fixed interval of time steps until the animal moved. After waiting the appropriate number of time steps, the animal moved to a new position in the landscape before the next time step (Figure 1b). The new location was chosen randomly by selecting a movement distance value from an exponential distribution with a mean distance (m) of one of six movement distance levels (see Section 2.3), and a randomly chosen direction from 0–360° from a uniform distribution (uncorrelated random walk with no directional tendency, Turchin, 1998).

Animal movement was permitted only within the bounds of the model landscape and among suitable habitat cells (Figure 1b), reflecting constraints faced by animals dependent upon one habitat type among unsuitable habitat in real landscapes affected by habitat loss, such as forest within farmland (e.g., Breitbach, Böehning‐Gaese, Laube, & Schleuning, 2012; Lenz et al., 2011). The animal could cross matrix cells, but only if the destination cell was in suitable habitat and not in matrix. If SEADS selected a movement location in a matrix cell, the new location was placed in the suitable habitat cell closest to the new matrix location that fell along a straight line from the previous to the new location. This simulates an animal moving as far as possible in the chosen direction from the previous position and ending movement at the edge of the last patch crossed before the chosen location in matrix. If a randomly selected movement ended outside the landscape, the movement was resampled until it fell within the landscape. We selected these rules to approximate movement of an animal for which (1) matrix habitat is semipermeable such that the likelihood of crossing a forest gap is a function of gap size, and (2) the number of movement events is not a function of habitat loss, e.g., is obligate for resource acquisition. Although some animals regard matrix portions of their habitat as suitable habitat for potential seed dispersal (e.g., Uriarte et al., 2011), SEADS focuses on the most straightforward scenario of suitable and nonsuitable habitat as a starting point to explore seed dispersal dynamics under varying levels of habitat loss.

#### 2.8.2 Seed dispersal

To incorporate the effects of gut retention times, each of the 100 seeds consumed by the animal at time step 0 were retained until an assigned exit time was reached during each simulation run. Exit times for each seed were randomly selected at the beginning of each simulation from a gamma distribution (see Section 2.3) and rounded to discrete 1‐min time steps. We desired the exit time distribution to be centered on one of the six treatment levels but also be skewed toward longer than average retention times. To create this effect, we used a gamma (shape = 4, scale = 5) that was shifted to the right. The extent of the rightward shift was chosen so that the mean of the shifted distribution equaled the selected treatment level (e.g., Levey, Tewksbury, & Bolker, 2008; Levey et al., 2005). When the exit time of each seed was reached, SEADS recorded the current location of the animal as the location of the seed (Figure 1c).

If seeds were dispersed in a time step between animal movements, the location of seed deposition was randomly varied within a 25 m² (5 × 5 cells) area around the location of the animal. This small‐scale seed movement helped simulate more realistic seed dispersal at the microsite and simplified the calculation of seed aggregation. We interpreted this variation as fine‐scale seed dispersal while the animal makes small movements of ≤2 m on or near a fruiting plant (e.g., Spiegel & Nathan, 2007). Seeds could only be dispersed at animal locations at the end of a time step and thus could not be dispersed in matrix cells during movement. Multiple seeds could be dispersed per time step. Each simulation was complete when all 100 seeds were dispersed.

For each simulation, one level of habitat loss was chosen and the disperser was assigned one value within the range for each animal trait. For each combination of the animal traits and habitat loss, 100 replicates of the model were run, for a total of 2,160 combinations of parameters and scenarios and 216,000 runs of the model. Data were then postprocessed to estimate dispersal distances and seed dispersion as a measure of aggregation and conduct statistical analyses.

#### 2.8.3 Dispersal distance and aggregation metrics

As a traditional metric of dispersal distance (e.g., Clark, Poulsen, Bolker, Connor, & Parker, 2005), the distance from the parent plant (start location) to each seed was calculated and averaged for each simulation run (100 seeds, Figure 1d). An established method to estimate seed aggregation is to calculate an index of the density of neighbor propagules within a distance class to the density of all propagules across the landscape (Caughlin et al., 2015; Condit et al., 2000). However, this index did not meet the assumptions of equal variance and linearity for our statistical analyses. Instead, this method was modified and our metric of seed aggregation was calculated as the mean distance (m) of seeds from the mean location of all seeds in each model run (Figure 1e), according to the following equation,

$$\text{SD}=\;\frac{\sum \surd {\left(x_{m}-x_{i}\right)}^{2}+{\left(y_{m}-y_{i}\right)}^{2}}{n}$$

where *n* is the number of seeds per model run (100), *x*_*m*_, *y*_*m*_ is the mean location of *n* seeds, and *x*_*i*_, *y*_*i*_ is the location of each individual seed. To avoid confusion, because this metric is a measure of distances from a spatial mean, we refer to it as seed dispersion for our model results instead of aggregation. Thus, depositing seeds more evenly across a landscape in this context would be increasing seed dispersion versus decreasing seed aggregation.

### 2.9 Statistical analyses

The influence of multiple parameters on dispersal distance and seed dispersion was simultaneously evaluated using multiple linear regression analyses to estimate the relative influence of the predictor variables (MD, GRT, TBM, habitat loss, and their interactions) on dispersal distance and seed dispersion. Because habitat loss showed a second‐degree polynomial trend for both dispersal distances and dispersion, a polynomial term was added to the linear regression model (HL²). The mean of the MD sampling distribution was used for statistical analyses instead of realized means from the stochastic simulations, the latter of which may have been confounded by habitat loss. Similarly, the mean of the GRT sampling distribution was used in the statistical analyses.

Because the concept of statistical significance has little meaning in simulations where high sample sizes can be easily obtained (up to 216,000 model runs in our case), enabling identification of extremely small differences between treatments, statistical analyses were conducted only to estimate effect sizes of the predictor variables and to assess their contribution to statistical model fit. Effect sizes were assessed based on regression coefficients for predictor variables (Quinn & Keough, 2002; Schielzeth, 2010). Each variable was first divided by its standard deviation to standardize them. Use of standardized variables made it possible to directly use regression coefficients as measures of effect size to compare their relative contribution to model fit (Quinn & Keough, 2002; Schielzeth, 2010).

Akaike information criterion was used to determine the most parsimonious statistical model for each analysis (Burnham & Anderson, 2002). Results for the most supported statistical models are reported and values for standardized regression coefficients are referred to as effect sizes. Adjusted r‐squared results are reported for the most supported models as an additional indicator of model fit to the data. Four analyses were conducted, one set with animal trait variables at 0% habitat loss with either dispersal distance or seed dispersion as the response variable, and another set that also included habitat loss as a predictor variable for both response variables. To determine whether our simulation results were sensitive to the size of the start patch or matrix gaps in our habitat loss scenarios (100 m²), we also ran simulations and conducted statistical analyses for models in which the start patch and matrix gap size were 50 and 200 m². All statistical analyses were conducted, and all figures were produced in R (R Development Core Team 2015).

## 3 RESULTS

### 3.1 0% habitat loss scenario

**Figure 4:**
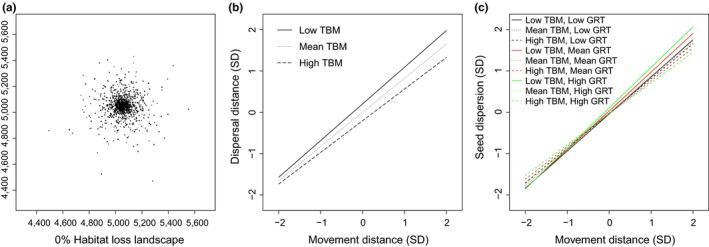
Spatial patterns of seeds dispersed by an animal in 0% habitat loss simulations (a), and results of regressions of standardized data under 0% habitat loss for the effects of the interaction of movement distance (MD) and time between movements on dispersal distance (b), and the effects of the interaction of MD, gut retention time (GRT), and time between movements (TBM) on seed dispersion (c). Units for the *x* and *y* axes are coordinates of locations in the theoretical landscape (a), or standard deviations from the mean (b, c). High, medium, and low levels for TBM (b) or TBM and GRT combinations (c) represent 2, 0, and −2 standard deviations from the mean

The majority of seeds were dispersed near and around the start point and radiated in decreasing frequency from it under the 0% habitat loss scenario (Figure 4a). For both dispersal distance and seed dispersion, movement distance had the highest effect size (0.83 and 0.87, respectively, Table 2). All other variables had effect sizes of 0.10 or lower (Table 2). The most supported statistical model included all three animal traits and an MD × TBM interaction for seed dispersal distance in the absence of habitat loss, representing 69% of the variation in the data (Table 3). Increasing MD and decreasing TBM resulted in increased dispersal distances (Figure 4b). Additionally, the difference between short and long TBM on dispersal distances became larger as MD increased (Figure 4b).

**Table 2:** Standardized regression coefficients from the most supported statistical models for multiple linear regression analyses of predictor variables on seed dispersal distance (Distance) and seed dispersion (Dispersion) in landscape scenarios of continuous habitat (0%) and over a range of habitat loss levels (0%–90%). Some variables were not applicable in some analyses and are represented by “‐”

Predictor variable	0% Habitat loss		0%–90% Habitat loss	
	Distance	Dispersion	Distance	Dispersion
Movement distance (MD)	0.83	0.87	0.86	0.91
Gut retention time (GRT)	0.01	0.02	0.14	0.13
Time between movement (TBM)	−0.10	−0.02	−0.20	−0.11
Habitat loss (HL)	–	–	0.01	0.01
HL²	–	–	−0.04	−0.05
MD × GRT	a	0.00^a^	0.10	0.10
MD × TBM	−0.03	−0.04	−0.10	−0.08
MD × HL	–	–	−0.02	−0.03
MD × HL²	–	–	−0.04	−0.07
GRT × TBM	a	−0.01	−0.05	−0.05
GRT × HL	–	–	0.04	0.04
GRT × HL²	–	–	−0.02	−0.01
TBM × HL	–	–	−0.03	−0.04
TBM × HL²	–	–	0.02	0.01
MD × GRT × TBM	a	−0.01	−0.03	−0.03
MD × GRT × HL	–	–	0.02	0.02
MD × GRT × HL²	–	–	−0.02	−0.02
MD × TBM × HL	–	–	−0.01	−0.03
MD × TBM × HL²	–	–	0.02	0.00
GRT × TBM × HL	–	–	−0.01	−0.01
GRT × TBM × HL²	–	–	0.01	0.01
MD × GRT × TBM × HL	–	–	0.00	−0.01
MD × GRT × TBM × HL²	–	–	0.01	0.00

a Not significant.

**Table 3:** Akaike information criterion (AIC) and adjusted r‐squared values for statistical models for dispersal distance (Dispersal) and seed dispersion (Dispersion) in 0% habitat loss scenarios. The most supported models are shown in bold

Model	Dispersal		Dispersion	
	Adj r²	AIC	Adj r²	AIC
MD + GRT + TBM + MD × TBM	**0.694**	**35,716**	0.753	31,126
MD + GRT + TBM + MD × GRT + MD × TBM	**0.694**	**35,718**	0.753	31,128
MD + GRT + TBM + MD × TBM + GRT × TBM	**0.694**	**35,718**	0.753	31,119
MD + GRT + TBM + MD × GRT + MD × TBM + GRT × TBM	0.694	35,720	0.753	31,121
Full model	0.694	35,722	**0.753**	**31,112**
MD + TBM + MD × TBM	0.694	35,724	0.752	31,149
MD + GRT + TBM	0.693	35,780	0.751	31,238
MD + GRT + TBM + MD × GRT	0.693	35,782	0.751	31,240
MD + GRT + TBM + GRT × TBM	0.693	35,782	0.752	31,232
MD + TBM	0.693	35,788	0.751	31,261
MD + GRT	0.683	36,498	0.751	31,259
MD + GRT + MD × GRT	0.683	36,500	0.751	31,261
MD	0.683	36,505	0.751	31,282
GRT + TBM	0.010	61,076	0.000	61,293
TBM	0.010	61,078	0.000	61,297
GRT + TBM + GRT × TBM	0.010	61,078	0.001	61,293
GRT	0.000	61,300	0.000	61,297
Null	0.000	61,301	0.000	61,301

In contrast to dispersal distance results, the most supported statistical model for seed dispersion in the 0% habitat loss scenario included the interaction of all three animal traits, representing 75% of the variation in the data (Table 3). Effect sizes were similar to results for dispersal distance, except that TBM was much less important for dispersion (Table 2). At low MD, the highest dispersion of seeds occurred when both TBM and GRT were high; however, the differences in dispersion among trait combinations were small. At the highest MD, dispersion increased with decreasing TBM (Figure 4c).

### 3.2 10%–90% habitat loss scenarios

Results for animal traits were consistent for scenarios with and without habitat loss; increasing MD and GRT and decreasing TBM increased dispersal distances overall. Movement distance was also the variable with the highest effect size for both dispersal distance and seed dispersion (0.86 and 0.91, respectively) under habitat loss scenarios, also similar to results for 0% habitat loss (Table 2). The full model, including a four‐way interaction of all three animal traits and habitat loss and its squared term, was the most supported statistical model for dispersal distance and seed dispersion under 0%–90% habitat loss scenarios, which explained 74% and 76% of the variation in the data, respectively (Table 4).

**Table 4:** Akaike information criterion (AIC) for select statistical models for dispersal distance (Dispersal) and seed dispersion (Dispersion) for simulations differing in start patch size. The base simulation is 100 m². Adjusted r‐squared values are for the most supported models, both shown in bold

Model	Patch size					
	50 m²	100 m²	200 m²	50 m²	100 m²	200 m²
	Dispersal AIC			Dispersion AIC		
Full model	**322,514**	**325,143**	**330,470**	**304,041**	**301,583**	**299,002**
Main effects + 2‐way interactions + 3‐way interactions + MD × GRT × TBM × HL	322,544	325,182	330,524	304,136	301,597	299,096
Main effects + 2‐way interactions + 3‐way interactions	322,548	325,197	330,524	304,149	301,637	299,107
Main effects + 2‐way interactions	324,566	327,158	331,887	306,832	303,964	301,311
MD + GRT + TBM + HL + HL²	344,994	343,556	341,105	329,498	324,430	311,307
MD + GRT + TBM + HL	347,392	344,610	341,402	332,583	326,301	311,732
MD + GRT + TBM	347,617	344,927	341,716	332,855	326,638	311,953
Null model	612,984	612,984	612,984	612,984	612,984	612,984
**Adjusted r‐squared**	**0.74**	**0.74**	**0.73**	**0.76**	**0.76**	**0.77**

**Figure 5:**
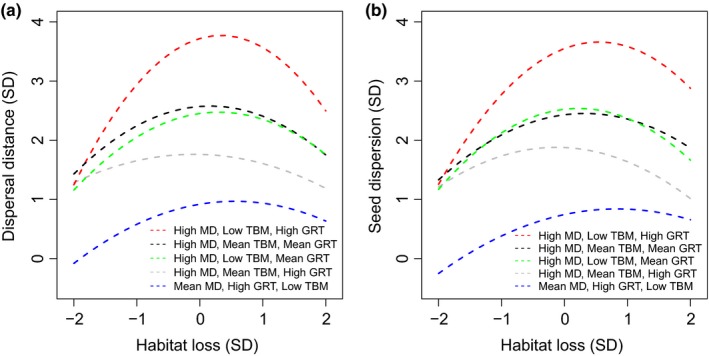
Combinations of movement distance (MD), gut retention time (GRT), and time between movements (TBM) on dispersal distance (a) and seed dispersion (b) for which habitat loss was nonlinear, based on regression analyses of standardized data. Units for the *x* and *y* axes are standard deviations from the mean. High, medium, and low levels for MD, TBM and GRT represent 2, 0, and −2 standard deviations from the mean

**Figure 6:**
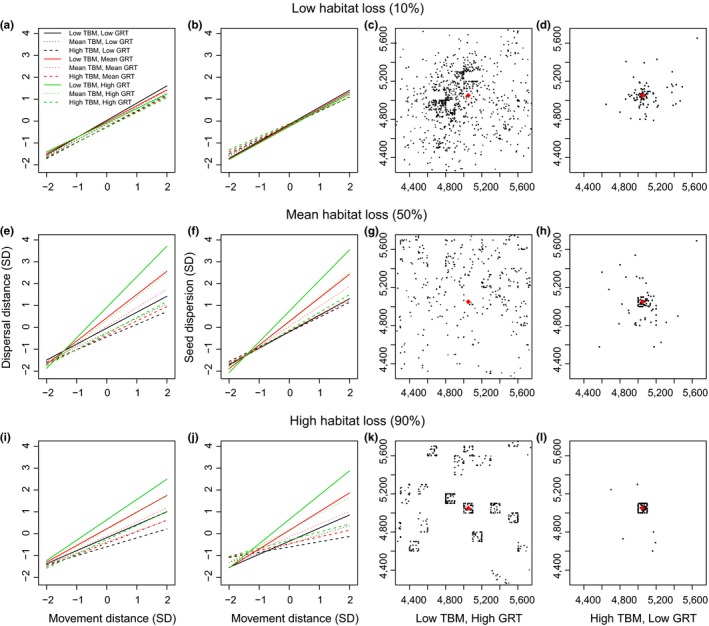
Regressions, using standardized data, to examine the interaction of movement distance (MD), gut retention time (GRT), and time between movements (TBM) on dispersal distance and seed dispersion at low (10%), mean (50%), and high (90%) habitat loss (left 2 columns of plots, a, b, e, f, i, and j). Spatial patterns of seeds dispersed by an animal in 0%–90% habitat loss simulations at the highest (low TBM, high GRT, c, g, and k) and lowest (high TBM, low GRT, d, h, and l) combinations of TBM and GRT for high MD (right 2 columns of plots, c, d, g, h, k, and l). Units for the *x* and *y* axes are standard deviations from the mean (left plots, a, b, e, f, i, and j) or coordinate locations in theoretical landscapes (right plots, c, d, g, h, k, and l). High, medium, and low levels for TBM and GRT combinations represent 2, 0, and −2 standard deviations from the mean. The parent tree in the middle of the start patch is represented by a red diamond

The four‐way interaction revealed a second‐degree polynomial trend of habitat loss for several combinations of animal traits for both dispersal distance (Figure 5a) and seed dispersion (Figure 5b). At low levels of habitat loss, both dispersal distances and dispersion increased as MD increased; however, combinations of TBM and GRT resulted in similar dispersal distances (Figure 6a) and seed dispersion (Figure 6b). Seed deposition patterns were more evenly spread across the landscape at low TBM and high GRT (Figure 6c) compared to high TBM and low GRT, in which seeds were deposited in localized aggregations (Figure 6d). However, in both scenarios, a large number of seeds were similarly centered in the middle of the landscape, on or near the start patch, despite the animal having few landscape barriers to movement (Figure 6c,d).

As MD increased at intermediate levels of habitat loss, some combinations of TBM and GRT (low TBM and high or mean GRT, mean TBM and high GRT) resulted in higher dispersal distances (Figures 5a and 6e) and seed dispersion (Figures 5b and 6f) than the same combinations at low levels of habitat loss (Figure 6a,b). Seed deposition patterns at high MD for low TBM and high GRT, the combination resulting in the highest dispersal distances and dispersion, were the most evenly spread across the landscape of all scenarios and showed little aggregation (Figure 6g). Conversely, other combinations of TBM and GRT led to decreased dispersal distances and dispersion (Figure 6h) relative to the same combinations at low habitat loss (Figure 6c,d). For high TBM and low GRT, resulting in the lowest dispersal distances and dispersion, some seeds were deposited throughout the landscape, but the majority of seeds were concentrated within the start patch (Figure 6h).

At high levels of habitat loss, most combinations of TBM and GRT resulted in lower dispersal distances (Figure 6i) and dispersion (Figure 6j), below the same combinations at low habitat loss (Figure 6a,b). This was particularly true for TBM and GRT combinations with low TBM, and for seed dispersion results (Figure 6j). At these combinations of TBM and GRT at high MD, a few seeds were deposited throughout the landscape; however, almost all were concentrated within the start patch (Figure 6l). However, higher levels of seed dispersal and dispersion occurred when MD and GRT were high and TBM was low when habitat loss was high (Figure 6i) than when it was low (Figure 6a,b). For this combination at high MD, high habitat loss fragmented the landscape into isolated patches; thus, seeds were deposited across many patches in the landscape, but were also locally aggregated within patches (Figure 6k).

**Figure 7:**
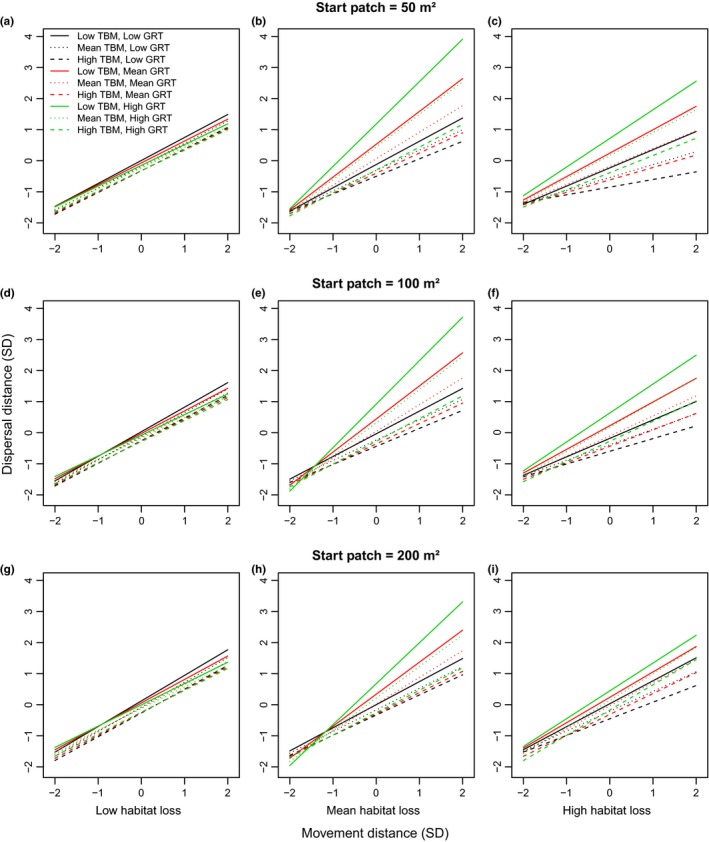
The interaction of movement distance, gut retention time (GRT), and time between movements (TBM) on dispersal distance at low (a,d,g), mean (b,e,h) and high habitat loss (c,f,i) based on regression analyses of standardized data. Units for the *x* and *y* axes are standard deviations from the mean. High, medium, and low levels for TBM and GRT represent 2, 0, and −2 standard deviations from the mean

**Figure 8:**
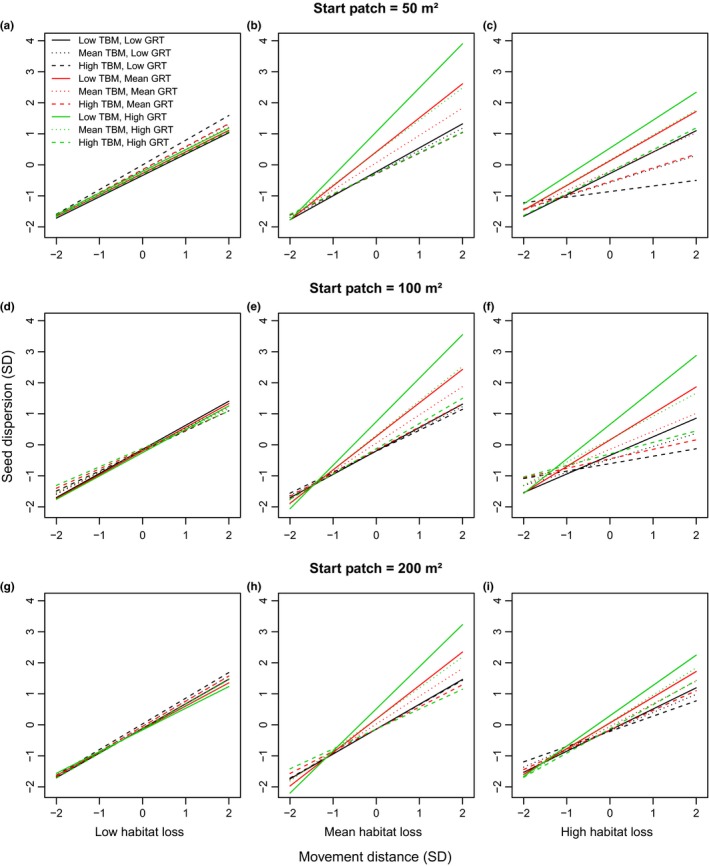
The interaction of movement distance, gut retention time (GRT), and time between movements (TBM) on seed dispersion at low (a,d,g) mean (b,e,h) and high habitat loss (c,f,i) based on regression analyses of standardized data. Units for the *x* and *y* axes are standard deviations from the mean. High, medium, and low levels for TBM and GRT represent 2, 0, and −2 standard deviations from the mean

Changing the size of the start patch and matrix patches altered the details of variation in dispersal distance and dispersion values in most habitat loss scenarios, but did not change the overall qualitative results of SEADS simulations. As with the base simulation (100 m² start and matrix patch size), the full models, including the four‐way interaction, were the most supported statistical models for both simulations with start patch and matrix patch sizes of 50 and 200 m² (Table 4). Effect sizes were similar among simulations differing in patch size (Appendix S1). At low habitat loss, results were similar for both dispersal distance (Figure 7a,d,g) and seed dispersion (Figure 8a,d,g), regardless of the size of the start and matrix patches or movement distance. However, at mean and high habitat loss, the difference between the effects of combinations of GRT and TBM at high MD was greatest when the size of the start and matrix patches was small (50 m²) compared to large (200 m²) for both dispersal distance (Figure 7b,c,e,f,h,i) and dispersion (Figure 8b,c,e,f,h,i). Thus, at high MD, for the low TBM and high GRT combination and the high TBM and low GRT combination, the model with the smallest patch sizes produced both the highest and lowest values, respectively, for dispersal distance (Figure 7) and dispersion (Figure 8) relative to models with medium (100 m²) and large (200 m²) patch sizes.

## 4 DISCUSSION

The spatial patterns of animal‐mediated seed dispersal can be as varied and complex as the behaviors and traits of animal vectors plus their environmental interactions (Côrtes & Uriarte, 2013; Karubian & Durães, 2009; Nathan & Muller‐Landau, 2000; Schupp et al., 2002). We found that spatial patterns of dispersal distances and seed dispersion, our metric of aggregation, were driven by a complex interaction involving all three animal traits and habitat loss. Low and high habitat loss reduced dispersal distances and seed dispersion by restricting animal movement. Unexpectedly, we also found that intermediate levels of habitat loss can increase dispersal distances and dispersion. However, whether the effects of habitat loss were positive or negative depended on the animal traits for movements and gut retention times of the disperser. Understanding the effects of these interactions with habitat loss on seed dispersal distances and seed aggregation is a critical step in closing knowledge and literal gaps for seed dispersal in disturbed landscapes (Karubian & Durães, 2009; Markl et al., 2012; McConkey et al., 2012).

### 4.1 Effects of animal traits in continuous habitat

Movement distance, compared to gut retention time and time between movements, was the most influential variable among animal traits in determining both dispersal distances and seed dispersion in 0% habitat loss simulations, supporting results from previous studies for dispersal distance (Murray et al., 1994; Rodríguez‐Pérez et al., 2012; Wotton & Kelly, 2012). If movement distances are short, our results indicate that increasing gut retention times or decreasing time between movements is ineffective at increasing dispersal distances or dispersion. For example, Baleric lizards (*Podarcis lilfordi*) dispersed *Ephedra fragilis* seeds an average of only 72 m from parent plants because they stayed within small territories, despite average gut retention times of 2 to 3 days (Rodríguez‐Pérez et al., 2012). Under 0% habitat loss scenarios, our results indicated that time between movements was secondarily important for both dispersal distances and dispersion. This concurs with studies in continuous rainforests; making more frequent movements during active foraging permitted southern cassowaries (*Casuarius casuarius*) and toucans to increase dispersal distances 1.4 and 2 times further, respectively, than average dispersal distances (Kays et al., 2011; Westcott et al., 2005). Although gut retention time was not influential at 0% habitat loss for dispersal distance, for seed dispersion, higher gut retention times would provide the animal with additional time to move more often and further, spreading seeds out more evenly across the landscape.

### 4.2 Habitat loss effects

SEADS simulations revealed the underlying qualitative mechanism by which habitat loss restricted animal movement, which typically reduced dispersal distance and dispersion in our landscape. Habitat loss created gaps in the landscape that influenced animal movement, often causing our disperser to become trapped in the start or other patches that permitted only short‐distance movements and increased time spent on edges. The disperser remained trapped within patches and often along patch edges until the model randomly selected a large enough movement distance from the movement distribution to allow it to traverse matrix gaps and escape to another suitable patch. If distances to nearby suitable habitat patches were high, the disperser was unlikely to move to a new patch within gut retention times. If unable to escape such patches, the animal dispersed most or all of its seeds in the same small area, resulting in aggregated dispersal, often within the parent patch. We label this underlying mechanism “fragment entrapment.”

Fragment entrapment played an important positive and negative role in determining seed dispersal distances and dispersion at different levels of habitat loss. In 0% or low habitat loss scenarios, little or no barriers existed in the landscape to impede animal movement. Because the direction of the animal disperser was chosen randomly at each movement interval in our simulations, the animal was just as likely to move backward toward the start point as move away from it (random walk with no directionality, Turchin, 1998). Thus, based on these random movements of the disperser from the start point within gut retention times, although many seeds were dispersed away from the parent plant in these scenarios, many were also dispersed near it as well, limiting dispersal distance and seed dispersion. For example, although red howler monkeys (*Alouatta seniculus*) in continuous forest in Columbia moved up to 1,875 m before dispersing seeds, some seeds were dispersed on average as little as 231 m from parent trees because the monkeys often moved in circular patterns within the same territorial area (Yumoto et al., 1999).

In contrast, at intermediate and high levels of habitat loss, if the animal crossed matrix to reach other nearby patches of suitable habitat, it often became trapped in the new patch and was less likely to cross matrix and return to the start or previous patch, which tended to increase dispersal distances and dispersion relative to random movements without barriers. At intermediate habitat loss, patches of suitable habitat were relatively close, and the animal often escaped the start patch, typically increasing dispersal distance and dispersion relative to 0% or low habitat loss scenarios. In contrast, at high levels of habitat loss, suitable patches were more isolated and further from the start patch than at intermediate distances, resulting in low dispersal distances and dispersion relative to intermediate habitat loss because the animal rarely escaped the start patch before dispersing seeds. One study on howler monkeys (*Alouatta palliata mexicana*) comparing seed dispersal in an isolated forest fragment to continuous forest in Mexico illustrates how fragment entrapment can decrease dispersal distances and dispersion (Serio‐Silva & Rico‐Gray, 2002). Monkeys in the fragment dispersed seeds in more aggregated patterns and closer to parent trees because groups visited the same fruiting trees multiple times compared to troops in continuous forest that visited most trees one time per fruiting season.

A study on pollen dispersal in a fragmented forest landscape in Costa Rica also supported our simulation results: forest gaps as small as 50 m restricted the movements of a generalist hummingbird pollinator, impeding dispersal among adjacent fragments (Volpe et al., 2016). In contrast to these results, studies from molecular analyses of animal‐mediated pollen dispersal in disturbed landscapes suggest that habitat loss does not affect or even increases dispersal distances (reviewed in Hamrick, 2010). However, the restriction of animal movements and fragment entrapment due to habitat loss within the limits of gut retention times could explain these effects for animal seed dispersal. If movement is constrained by matrix gaps on one or more sides, an animal may have difficulty leaving an area to disperse seeds long distances before seeds exit the animal. Pollen detachment from dispersers, in contrast, is not limited by this time constraint (Wheelwright & Orians, 1982). Thus, even if long‐distance movements are rare because pollen dispersers rarely escape to adjacent fragments, such movements could result in successful dispersal among patches if pollen remains on the disperser for long periods of time.

### 4.3 Mitigating effects of animal traits on habitat loss

The interactions of animal traits determined whether fragment entrapment resulted in higher or lower dispersal distances and seed dispersion relative to 0% or low habitat loss scenarios. Movement distance was also the most influential animal trait mitigating the negative effects of habitat loss on both dispersal distances and dispersion in our simulations. High values for movement distance increased the likelihood of the animal crossing matrix gaps and reaching suitable patches outside the start patch. However, at low values of movement distance, the animal was unlikely to escape the start patch, resulting in low dispersal distances and seed dispersion as the animal deposited seeds near the parent plant and in aggregated spatial patterns. Similarly, in the literature, the short‐distance movements of common blackbirds (*Turdus merula*) in Germany were predicted to disperse only 14.9% of cherry seeds (*Prunus avium*) > 100 m in fragmented farmland compared to 28.2% in continuous forest (Breitbach et al., 2012). In contrast, the long‐distance movements of trumpeter hornbills (*Bycanistes bucinator*) in South Africa (up to 15 km) facilitated seed dispersal among over 100 forest patches, doubling the functional connectivity of a heavily fragmented forest landscape (Mueller, Lenz, Caprano, Fiedler, & Böhning‐Gaese, 2014).

Secondary to movement distance, combinations of high gut retention time and low time between movements typically improved dispersal distance and dispersion values in our simulations because they provide additional time and movement opportunities to escape fragment entrapment. However, if a matrix gap was too large for the disperser to cross and reach adjacent areas, increasing gut retention time or decreasing time between movements could do little to improve dispersal distances, although decreasing time between movements improved seed dispersion within patches. Our results for animal traits agree with patterns in the literature suggesting that body size may be a simple proxy to assess the potential of a disperser to mitigate the effects of habitat loss, because larger animals tend to exhibit high movement distances and gut retention times (Wotton & Kelly, 2012; table 1). However, our results also indicate that regardless of body size, dispersers moving often (low time between movements) may be somewhat effective, particularly for seed dispersion. Furthermore, large animals are less likely to persist as functional dispersers in disturbed landscapes as habitat loss increases (reviewed in Markl et al., 2012; McConkey et al., 2012). In systems where effective large (e.g., Moura, Cavalcanti, Leite‐Filho, Mesquita, & McConkey, 2015) or native (e.g., Wu, Delparte, & Hart, 2014) dispersers have disappeared or are functionally absent (e.g., McConkey & Drake, 2006), understanding which traits or trait combinations lead to successful seed dispersal can aid in identifying potential complementary (e.g., Spiegel & Nathan, 2007), redundant (e.g., Uriarte et al., 2011), or replacement (e.g., Moura et al., 2015) dispersers.

Interactions between the size of start and matrix patches also determined the effectiveness of animal traits in overcoming the negative consequences of habitat loss on dispersal distance and dispersion. As the size of the start patch and matrix gaps increased, the variation in dispersal distance and dispersion results from different combinations of animal traits decreased. In small start patches (50 m²), a higher number of combinations of animal traits permitted the animal to escape the start patch more often relative to larger start patches (100, 200 m²), increasing relative dispersal distance and dispersion. However, when a combination of animal traits resulted in trapping the animal within a small start patch, dispersal distances and dispersion were lower than for simulations with larger start patches. These results mirrored dispersal distances by avian dispersers in the Brazilian Amazon; only one of six passerine species contributed to long‐distance dispersal events of an understory herb from 1 ha forest fragments because it was able to move to other fragments (Uriarte et al., 2011).

## 5 CONCLUSIONS

Quantitatively comparing which plant, animal, and landscape interactions are most important in creating the spatial patterns of seed dispersal, particularly in disturbed ecosystems, is critical to advancing theory and informing conservationists (Côrtes & Uriarte, 2013; Cousens et al., 2010; Nathan & Muller‐Landau, 2000). To our knowledge, this study is the first to quantify the effects of animal traits and landscape factors on spatial patterns of seed aggregation, which has important implications for seed mortality and plant populations (Caughlin et al., 2015; Harrison et al., 2013; Schupp et al., 2002). More importantly, we found that habitat loss had unexpected, nonlinear responses on seed dispersal and dispersion for several combinations of animal traits, driven by the novel mechanism of fragment entrapment. Results of our SEADS simulations can be generalized to other systems affected by habitat loss and applied as starting points for empirical testing. Studies comparing two levels of habitat loss will detect only a portion of the relationship between habitat loss and spatial patterns of seed dispersal. For example, studies that compare intermediate to high habitat loss sites might detect reduced dispersal distances and dispersion, whereas comparisons of continuous habitat to sites of intermediate habitat loss might detect the opposite pattern. Furthermore, the combination of animal traits is likely to influence the outcome of these comparisons. Assessment of our findings would thus benefit from a study design that incorporated multiple levels of habitat loss. However, only a few studies have quantified seed dispersal distances among multiple fragments (e.g., Lenz et al., 2011; McEuen & Curran, 2004; Uriarte et al., 2011). Mechanistic modeling of seed dispersal by animals is a powerful approach to quantify spatial patterns of seed dispersal and their drivers (Côrtes & Uriarte, 2013; Cousens et al., 2010; Nathan & Muller‐Landau, 2000), to close the knowledge and literal gaps in landscapes affected by habitat loss.

## AUTHOR CONTRIBUTIONS

LJ and DJ conceived and designed the modeling simulations. LJ analyzed the data and wrote the manuscript. SDS contributed to model construction, statistical analysis, and manuscript editing. PL contributed to data analysis and manuscript editing. DJ also contributed to manuscript editing.

## CONFLICTS OF INTEREST

None declared.
