# Supplementary material for: Closing the gaps for animal seed dispersal: Separating the effects of habitat loss on dispersal distances and seed aggregation
Source: Ecol Evol. 2017 Jun 12;7(14):5410–25. doi: 10.1002/ece3.3113 (PMC5528214; doi:10.1002/ece3.3113)
Supplement: Supplementary file 1 [file ECE3-7-5410-s001.docx]

**Closing the Gaps for Animal Seed Dispersal: Separating the Effects of Habitat Loss on Dispersal Distances and Seed Aggregation**

Landon R. Jones^1,*^, Scott M. Duke-Sylvester^1^, Paul L. Leberg^1^, and Derek M. Johnson^2^

*^1^ Department of Biology, 410 E. St. Mary Blvd, University of Louisiana at Lafayette, Lafayette, LA 70503, U.S.A.*

*^2^ Department of Biology, 1000 W. Cary St., Virginia Commonwealth University, Richmond, VA 23284, U.S.A.*

*^*^Corresponding author; email:* [*lrj1327@louisiana.edu*](mailto:lrj1327@louisiana.edu)*,*

**Supporting Information: Appendices**

**Appendices**

**Appendix S1**

Standardized regression coefficients representing the most supported statistical models for multiple linear regression analyses of predictor variables on seed dispersal distance (Distance) and seed dispersion (Dispersion) for simulations of varying sizes of start patches.

|  |  |  | **Start Patch Size** | |  |  |
| --- | --- | --- | --- | --- | --- | --- |
|  | **50 m²** | **100 m²** | **200 m²** | **50 m²** | **100 m²** | **200 m²** |
| **Predictor Variable** | **Distance** | | | **Dispersion** | | |
| movement distance (MD) | 0.85 | 0.86 | 0.86 | 0.88 | 0.91 | 0.90 |
| gut retention time (GRT) | 0.19 | 0.14 | 0.09 | 0.16 | 0.13 | 0.08 |
| time between movement (TBM) | -0.23 | -0.20 | -0.16 | -0.17 | -0.11 | -0.08 |
| habitat loss (HL) | 0.00 | 0.01 | 0.02 | 0.00 | 0.01 | 0.01 |
| HL² | -0.06 | -0.04 | -0.02 | -0.07 | -0.05 | -0.02 |
| MD x GRT | 0.10 | 0.10 | 0.08 | 0.08 | 0.10 | 0.05 |
| MD x TBM | -0.10 | -0.10 | -0.09 | -0.11 | -0.08 | -0.09 |
| MD x HL | -0.03 | -0.02 | 0.00 | -0.02 | -0.03 | -0.01 |
| MD x HL² | -0.05 | -0.04 | -0.03 | -0.05 | -0.07 | -0.04 |
| GRT x TBM | -0.07 | -0.05 | -0.04 | -0.08 | -0.05 | -0.04 |
| GRT x HL | 0.05 | 0.04 | 0.02 | 0.05 | 0.04 | 0.02 |
| GRT x HL² | -0.03 | -0.02 | -0.01 | -0.02 | -0.01 | -0.02 |
| TBM x HL | -0.04 | -0.03 | -0.01 | -0.05 | -0.04 | -0.02 |
| TBM x HL² | 0.02 | 0.02 | 0.01 | 0.03 | 0.01 | 0.02 |
| MD x GRT x TBM | -0.03 | -0.03 | -0.03 | -0.04 | -0.03 | -0.04 |
| MD x GRT x HL | 0.02 | 0.02 | 0.02 | 0.03 | 0.02 | 0.02 |
| MD x GRT x HL² | -0.02 | -0.02 | -0.02 | -0.01 | -0.02 | -0.01 |
| MD x TBM x HL | -0.02 | -0.01 | -0.01 | -0.02 | -0.03 | -0.02 |
| MD x TBM x HL² | 0.01 | 0.02 | 0.02 | 0.02 | 0.00 | 0.02 |
| GRT x TBM x HL | -0.01 | -0.01 | 0.00 | 0.00 | -0.01 | 0.00 |
| GRT x TBM x HL² | 0.01 | 0.01 | 0.01 | 0.02 | 0.01 | 0.01 |
| MD x GRT x TBM x HL | 0.00 | 0.00 | 0.00 | 0.01 | -0.01 | 0.00 |
| MD x GRT x TBM x HL² | 0.01 | 0.01 | 0.01 | 0.01 | 0.00 | 0.01 |

**Appendix S2**

Literature references for body mass of animal dispersers for which this information was not given in studies that reported three animal traits of seed dispersal (movement distance, gut retention time, time between movements, see Table 1).

|  |  |  |  |
| --- | --- | --- | --- |
| **Animal** | **Mass (kg)** | **Study Reference** | **Body Mass Reference** |
| bird | 0.030 | Levey et al. 2005, Levey et al. 2008 | Gowaty and Plissner 1998 |
| bird | 0.033 | Murray 1988 | Stiles and Skutch 1989 |
| bird | 0.056 | Murray 1988 | Stiles and Skutch 1989 |
| bird | 0.062 | Murray 1988 | Stiles and Skutch 1989 |
| mammal | 0.884 | Hickey et al. 1999 | Reid 2006 |
| mammal | 5.4 | Yumoto et al. 1999 | Boubli et al. 2015 |
| mammal | 7.4 | Yumoto et al. 1999 | Palacios et al. 2008 |
| reptile | 8.2 | Jerozolimski et al. 2009 | Morcatty and Valsecchi 2015 |

**References**

Boubli J-P, Di Fiore A, Rylands AB, Mittermeier RA (2015) *Alouatta seniculus* ssp. puruensis. The IUCN Red List of Threatened Species 2015

Gowaty PA, Plissner JH (1998) Eastern Bluebird (*Sialia sialis*). The Birds of North America:32.

Morcatty TQ, Valsecchi J (2015) Social, biological, and environmental drivers of the hunting and trade of the endangered yellow-footed tortoise in the Amazon. Ecol. Soc. 20:3.

Palacios E, Boubli J-P, Stevenson P, Di Fiore A, de la Torre S (2008) *Lagothrix lagotricha.* The IUCN Red List of Threatened Species 2008

Reid F (2006) A field guide to mammals of North America, north of Mexico. Houghton Mifflin Harcourt, Boston, MA

Stiles FG, Skutch AF (1989) Guide to the birds of Costa Rica. Comstock, Ithaca, NY
